# Proteomic Analysis of Human Macrophages Overexpressing Angiotensin-Converting Enzyme

**DOI:** 10.3390/ijms25137055

**Published:** 2024-06-27

**Authors:** Delia Oosthuizen, Tariq A. Ganief, Kenneth E. Bernstein, Edward D. Sturrock

**Affiliations:** 1 Division of Chemical, Systems and Synthetic Biology, Faculty of Health Sciences, Institute for Infectious Disease and Molecular Medicine, University of Cape Town, Observatory 7925, South Africa; 2 Department of Biomedical Sciences, Cedars-Sinai Medical Center, 8700 Beverly Blvd., Los Angeles, CA 90048, USA; 3 Department of Pathology and Laboratory Medicine, Cedars-Sinai Medical Center, 8700 Beverly Blvd., Los Angeles, CA 90048, USA

**Keywords:** angiotensin converting enzyme, discovery proteomics, ACE overexpression, myeloid cell, immunity, proteases, ACE inhibitors, macrophages

## Abstract

Angiotensin converting enzyme (ACE) exerts strong modulation of myeloid cell function independently of its cardiovascular arm. The success of the ACE-overexpressing murine macrophage model, ACE 10/10, in treating microbial infections and cancer opens a new avenue into whether ACE overexpression in human macrophages shares these benefits. Additionally, as ACE inhibitors are a widely used antihypertensive medication, their impact on ACE expressing immune cells is of interest and currently understudied. In the present study, we utilized mass spectrometry to characterize and assess global proteomic changes in an ACE-overexpressing human THP-1 cell line. Additionally, proteomic changes and cellular uptake following treatment with an ACE C-domain selective inhibitor, lisinopril–tryptophan, were also assessed. ACE activity was significantly reduced following inhibitor treatment, despite limited uptake within the cell, and both RNA processing and immune pathways were significantly dysregulated with treatment. Also present were upregulated energy and TCA cycle proteins and dysregulated cytokine and interleukin signaling proteins with ACE overexpression. A novel, functionally enriched immune pathway that appeared both with ACE overexpression and inhibitor treatment was neutrophil degranulation. ACE overexpression within human macrophages showed similarities with ACE 10/10 murine macrophages, paving the way for mechanistic studies aimed at understanding the altered immune function.

## 1. Introduction

Angiotensin converting enzyme (ACE) is a key regulator within the renin–angiotensin–aldosterone system (RAAS) [1,2,3], where it acts as a dipeptidase with two catalytically active domains, the N- and C-domains [4,5,6,7,8,9]. ACE has several substrates and it exerts influence over numerous biological pathways. Although well-known for its interaction with angiotensin I (Ang I), ACE exerts most of its hypertensive effects through its cleavage product angiotensin II (Ang II) and the angiotensin type I (AT1R) and type II receptors (AT2R) [10,11,12,13]. ACE also affects an important regulatory arm in the immune system, where it exerts metabolic changes in several important immune cell types [14,15], including macrophages and neutrophils, otherwise known as myeloid cells. Myeloid cells are pivotal to the immune response, as they ingest and destroy both infectious material and cellular debris, thus preventing these agents from causing further harm to the body [16,17,18].

ACE immune-related effects were initially thought to be mediated through Ang II, but subsequent transgenic murine ACE domain knockout and inhibition studies have shown this to be partially incorrect [19,20]. In fact, ACE overexpression in murine models has hinted at an Ang-II-independent improved immune state, particularly in the ACE 10/10 and NeuACE models which express high levels of ACE in macrophages and neutrophils, respectively [16,21,22,23,24].

ACE 10/10 mice show significantly improved resistance to atherosclerosis [25,26,27], cancer progression [21,28], Alzheimer’s disease [29,30,31,32], and bacterial infections [33]. Known metabolic changes resulting from ACE overexpression include increased lipid oxidation and ATP generation [34], increased peroxisome proliferator-activated receptor α (PPARα) expression [26,35], and enhanced major histocompatibility complex (MHC) I and II peptide creation [36,37,38]. These alterations are independent of Ang II and other known ACE substrates, implying the existence of a previously unknown peptide and biological pathway. Bernstein et al. have published in-depth reviews of the ACE 10/10 mouse model and its associated phenotype [14,16]. ACE is expressed in both human and murine macrophages, but global and targeted mass spectrometry studies have, to date, only focused on mouse models. Further studies in human cell line models are therefore required to ascertain if similar changes occur in human cells.

A human pilot study observed decreased resistance of human neutrophils toward bacterial infection after short-term ACE inhibitor (ACEi) usage, but not angiotensin receptor blockers (ARBs) [39]. Both intracellular and extracellular bactericidal mechanisms were negatively impacted by ACEi administration, with no inherent antimicrobial activity of the drugs lisinopril and ramipril observed. As both drugs bind either ACE domain, the reported reduced superoxide and reactive oxygen species (ROS) production in neutrophils could not be attributed to a specific domain. Importantly, domain-selective ACEis are currently in development to reduce side effects during hypertension treatment and allow domain-specific functions in relation to its wide substrate variety [40,41,42]. Since mouse models indicate that the ACE C-domain is required for enhanced immune effects [21], the mechanism by which ACE C-domain inhibitors impact macrophage and neutrophil function may be a key finding. However, there is a lack of experimental data on the effects of domain specific ACEis, including their unintentional uptake, in systems where ACE is also expressed.

During differentiation, human macrophages can increase their ACE expression by up to nine-fold following stimulation of a macrophage from a monocyte [43,44,45]. Understanding how the murine and human global macrophage proteome changes with altered ACE expression requires further investigation. Altering ACE expression and its resulting immune stimulation provides an exciting opportunity for alternative immunotherapy and treatment of infections, cancer, and other metabolic disorders. As increased ACE expression may enhance several immune functions, understanding the changes in the global murine and human macrophage proteome and the role of each domain is of great value. We therefore aimed to obtain preliminary global proteomics data in ACE-overexpressing human macrophages for the first time. These macrophages belong to the human THP-1 cell line and have been modified to overexpress full-length somatic ACE [27] to induce similar metabolic changes as in ACE 10/10 murine macrophages.

## 2. Results

### 2.1. Intracellular Uptake of ACE C-Domain Inhibitor, Lisinopril–Tryptophan, by Human THP-1 Macrophages

**Figure 1:**
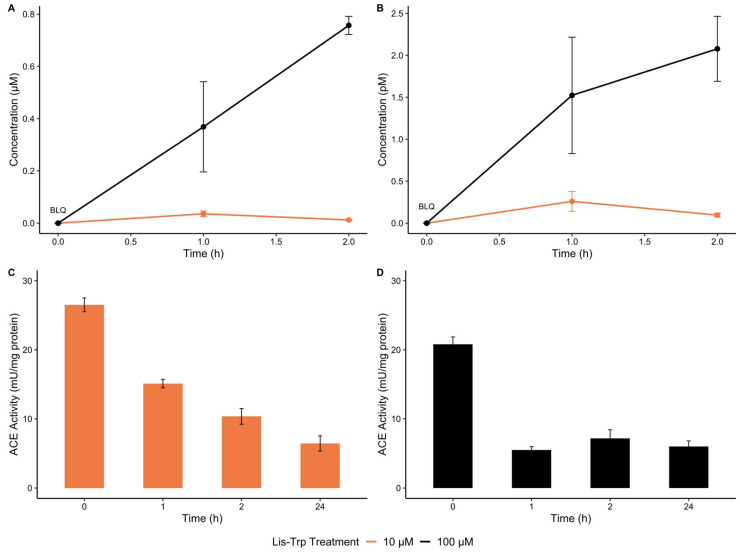
(**A**) The total Lis–Trp concentration (µM) detected in THP-1 macrophage lysate. (**B**) Lis–Trp intracellular concentrations (pM) normalized to total cell number. (**C**) ACE activity with 10 µM Lis–Trp treatment and (**D**) 100 µM Lis–Trp treatment. Total ACE activity (mU) was normalized against lysate mg protein. BLQ—below limit of quantitation, 0.00625 µM.

To confirm that domain-selective ACEis are capable of macrophage entry, an in vitro uptake assessment was performed (Figure S1). The C-domain ACEi, lisinopril–tryptophan (Lis–Trp), was measured hourly in lysed phorbol 12-myristate 13-acetate (PMA) stimulated THP-1 macrophages via mass spectrometry at treatment concentrations of 10 µM and 100 µM. Peak intracellular Lis–Trp concentrations were observed 1 h post-treatment in cells receiving 10 µM, but continued on an upward trend after 2 h in cells receiving 100 µM (Figure 1A,B). A higher dosage resulted in a higher drug uptake. However, no cellular stress, as determined by mass detachment or by Trypan Blue staining, was observed for either treatment.

Furthermore, ACE activity continuously decreased with ACEi treatment (Figure 1C,D). Less than 1% of Lis–Trp entered the THP-1 cells, but ACE activity was significantly impacted, with partial inhibition caused by both treatments.

### 2.2. Assessment of ACE +/+ THP-1 Macrophage Proteome

A total of 2317 proteins were identified and quantified, resulting in 2208 proteins after data filtering (Figure S1). Principle component analysis (PCA) revealed a clear grouping of ACE +/+ control samples, whereas control and ACEi-treated WT samples clustered together, indicating similar proteomic profiles (Figure S2). Lis–Trp-treated ACE +/+ THP-1 macrophage samples had greater variance between biological replicate samples.

#### 2.2.1. Differential Expression Analysis

**Figure 2:**
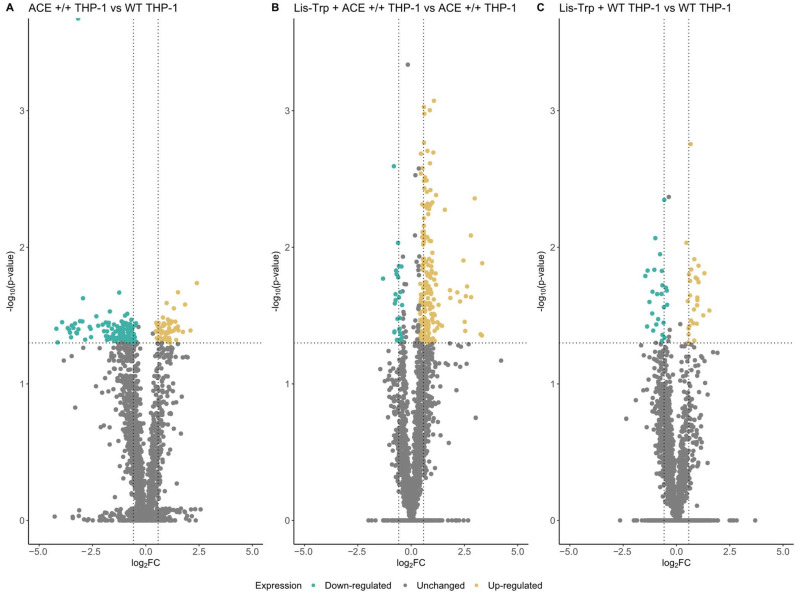
Significantly differentially expressed proteins (DEPs) across (**A**) ACE +/+ THP-1 compared to WT THP-1 macrophages, (**B**) untreated ACE +/+ THP-1 and Lis–Trp-treated ACE +/+ THP-1 macrophages, and (**C**) untreated WT THP-1 and Lis–Trp-treated WT THP-1 macrophages. A *p*-value ≤ 0.05 and log2FC ≥ |1.5| were regarded as significant. Blue represents downregulation and yellow represents upregulation.

Protein expression fold changes in the ACE +/+ THP-1 cell line were compared with the WT THP-1 cell line and Lis–Trp-treated samples with their corresponding untreated controls, applying a log2FC ≥ |1.5|and *p* ≤ 0.05 as cutoff. A total of 178 (55 upregulated and 123 downregulated) differentially expressed proteins (DEPs) were identified between ACE +/+ and WT THP-1 macrophages (Figure 2A). ACE expression was detected in all ACE +/+ samples, but it was absent in all WT THP-1 samples barring two (Figure S3), suggesting increased ACE expression in ACE +/+ THP-1 macrophages. This was consistent with western blot results of the ACE +/+ THP-1 cell line/THP-1^ACE^ in lipid-rich culture conditions [27]. Lis–Trp treatment of ACE +/+ and WT THP-1 macrophages resulted in 168 (153 upregulated and 15 downregulated) (Figure 2B) and 42 (22 upregulated and 20 downregulated) (Figure 2C) DEPs in comparison with control ACE +/+ and WT THP-1 macrophages, respectively, suggesting ACE inhibition had a greater effect on dysregulating protein expression than ACE overexpression.

#### 2.2.2. Gene Ontology (GO) Term Enrichment Analysis and Protein–Protein Interaction (PPI) Networks

Gene ontology (GO) term enrichment was applied to the DEPs of each comparison to identify overrepresented biological processes (BP). GO annotation results were filtered using a false discovery rate (FDR) ≤ 0.05 cutoff. Protein–protein interaction (PPI) networks were generated for the DEPs using a Markov cluster algorithm (MCL) to group interacting nodes (proteins) together. Interactions with a 0.4 confidence score and above are shown with a line thickness indicating degree of confidence.

**Figure 3:**
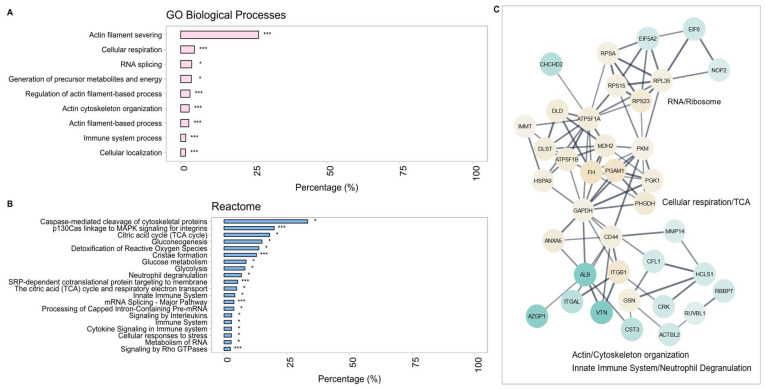
(**A**) Significantly enriched gene ontology (GO) biological process (BP) terms and (**B**) reactome pathways. (**C**) Protein–protein interaction (PPI) clusters within ACE +/+ THP-1 macrophages. A confidence score of 0.4 was applied with continuous color mapping indicating expression level. Blue = downregulation and yellow = upregulation in the ACE +/+ THP-1 group. The number of proteins in each process is given as a percentage (* *p* < 0.05, *** *p* < 0.0001).

An overall PPI enrichment value of *p* = 3.20 × 10^−11^ demonstrated significant interactions within the network generated using the DEPs of ACE +/+ and WT THP-1 macrophages. Given previous murine data suggesting that ACE overexpression is associated with immune function [16,21,28,36,38], it is encouraging that GO analysis identified the pathways ‘cellular respiration’ and ‘immune system process’ as highly significantly enriched in our data (Figure 3A). Additional reactome-based GO analyses generated several significantly enriched (FDR ≤ 0.05) terms specifically referring to ‘immune system’, ‘innate immune system’, immune signaling-related pathways for interleukins (IL) and cytokines, ‘glycolysis’, and the ‘citric acid/TCA cycle’ (Figure 3B). Proteins associated with these functions were largely upregulated in ACE-overexpressing THP-1 macrophages. The three most prominent of the 33 clusters pertaining to RNA metabolism, cellular respiration, and cytoskeletal organization are shown in Figure 3C. RNA metabolism, specifically mRNA splicing and mRNA processing, and cytoskeletal organization have not previously been associated with non-classical or classical ACE function, whilst upregulated cellular respiration has been associated with ACE overexpression in murine macrophages [34]. Of interest was the presence of the mitogen-activated protein kinase (MAPK) activation term: ‘p130Cas linkage to MAPK signaling for integrins’ with associated DEPs upregulated in the ACE +/+ THP-1 macrophages since ACE can act as a signaling molecule. Additionally, ‘neutrophil degranulation’ and ‘Rho GTPase signaling’ were also functionally enriched within this dataset and have not previously been identified in ACE 10/10 murine macrophages as an area of interest.

**Figure 4:**
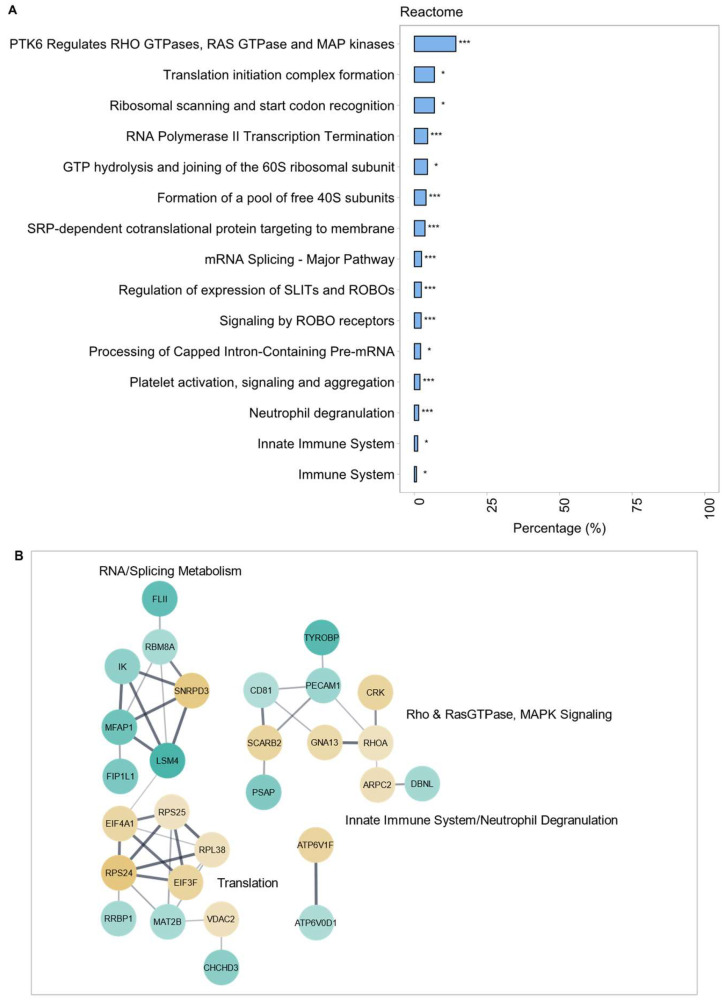
(**A**) Reactome pathways that were significantly functionally enriched. (**B**) Protein–protein interaction (PPI) clusters within the network of differentially expressed proteins (DEPs) after Lis–Trp treatment of WT THP-1 macrophages compared to untreated WT THP-1 macrophages. A confidence score of 0.4 was applied, with continuous color mapping indicating the expression level. Blue = downregulation and yellow = upregulation in the treated WT THP-1 group. The number of proteins present in each process is represented as a percentage (* *p* < 0.05, *** *p* < 0.0001).

Although WT THP-1 macrophages did not express high levels of ACE (Figure S3), the impact of Lis–Trp treatment was assessed, as basal ACE was still active and its inhibition may be evident in downstream functions. As expected, ACE inhibition of WT THP-1 macrophages had limited cellular signaling effect, as evidenced by the total number of DEPs. Functional enrichment analysis reactome GO-based terms (Figure 4A) and a PPI network (Figure 4B, *p* = 0.0026) composed of five functional clusters highlighted RNA metabolism as significantly enriched. These terms centered around RNA processing, translation, and modification such as splicing. Also significantly enriched were the terms ‘immune system’, ‘innate immune system’, and ‘neutrophil degranulation’, as they are all present in ACE +/+ THP-1 macrophages. Despite sharing the same functionally enriched terms and similar proteins, for example ribosomal protein S-components RPS24 and RPS25, ACE-inhibited WT THP-1 macrophages seemingly experienced more active translation initiation than ACE-overexpressing human macrophages. However, there also appeared to be increased ‘L13-a mediated silencing of ceruloplasmin expression’ and reduced RNA metabolism (‘RBM8A’, ‘IK’, ‘MFPA1’ and ‘LSM4’) with Lis–Trp treatment of WT THP-1 macrophages in comparison to ACE +/+ THP-1 macrophages. Interestingly, the immune functions were also seemingly less active with ACE inhibition in WT human macrophages, including the highly significantly enriched ‘neutrophil degranulation’ term with downregulated proteins ‘TYROBP’, ‘PECAM1’, ‘PSAP’, and ‘DBNL’ that were similarly negatively dysregulated in ACE +/+ THP-1 macrophages in comparison to WT THP-1 macrophages. There was also a lack of ‘cellular respiration’ and energy metabolism differential protein expression in the ACE-inhibited WT THP-1 macrophages, further adding to a link between increased ACE and increased oxidative and ATP metabolism-associated protein expression/activity.

**Figure 5:**
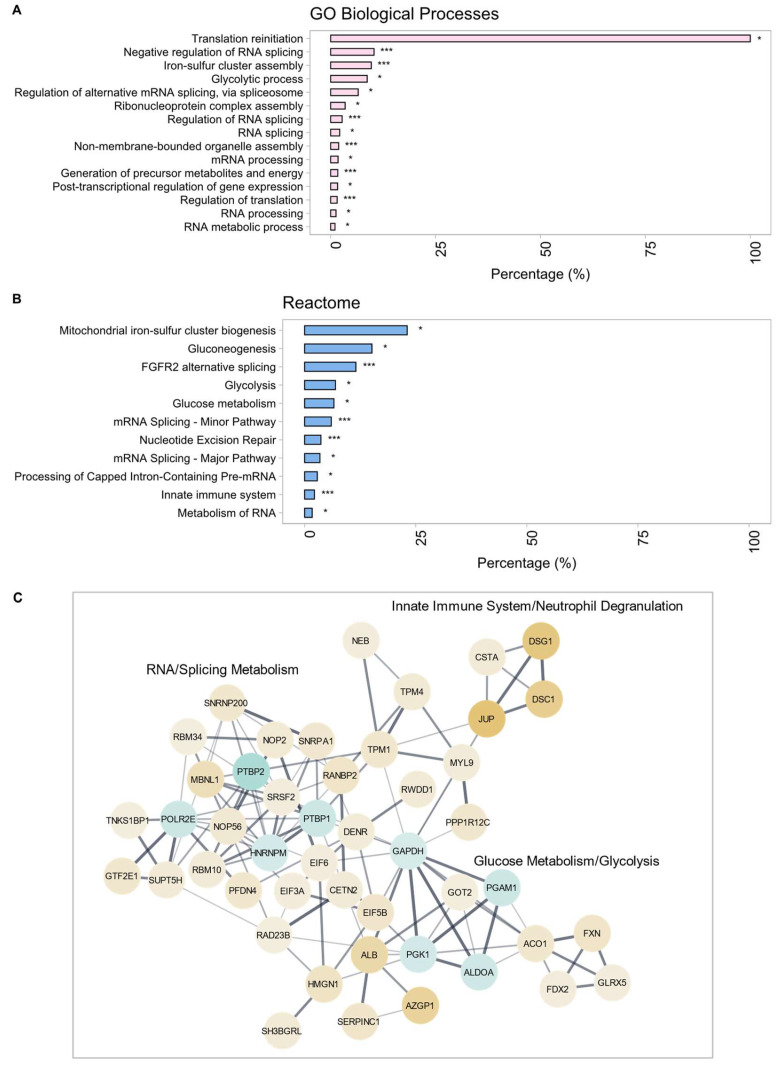
(**A**) Gene ontology (GO) terms relating to biological processes (BP) and (**B**) reactome pathways. (**C**) Protein–protein interaction (PPI) clusters selected from networks of DEPs identified following the Lis–Trp treatment of ACE +/+ THP-1 macrophages compared to untreated cells. A confidence score of 0.4 was applied with continuous color mapping indicating the expression level. Blue = downregulation and yellow = upregulation in the treated ACE +/+ THP-1 group compared to the untreated group. The number of proteins present in each process is represented as a percentage (* *p* < 0.05, *** *p* < 0.0001).

After Lis–Trp administration to ACE +/+ THP-1 macrophages, significant GO BP functional enrichment terms (Figure 5A) included: ‘glycolytic process’, ‘RNA metabolic process’, ‘RNA splicing’, and ‘generation of precursor metabolites and energy’, and significantly enriched reactome terms included: ‘glycolysis’, ‘gluconeogenesis’, ‘mRNA splicing—major pathway’, and ‘mRNA splicing—minor pathway’ (Figure 5B). The PPI network of Lis–Trp-treated ACE-overexpressing THP-1 macrophages (*p* = 4.22 × 10^−15^) with interactor clusters (three of 24) corresponding to ‘RNA splicing’, ‘metabolism of RNA’, and ‘gene expression’, ‘glycolysis’, ‘glucose metabolism’, and ‘neutrophil degranulation’ is shown in Figure 5C. Whilst proteins associated with the terms ‘mRNA splicing’ and other RNA metabolic terms appeared to be predominantly upregulated after the Lis–Trp treatment, the ACE +/+ THP-1 cell line appeared to experience significant downregulation of proteins associated with glucose metabolism, including the glycolytic proteins GAPDH, ALDOA, PGAM1, and PKG1 following the Lis–Trp treatment. The term ‘neutrophil degranulation’ was significantly enriched after Lis–Trp administration to ACE +/+ THP-1 macrophages, and the associated proteins, including ADAM10, NHLR3, PRSS3, AMP03, SERPNB12, TYROBP, JUP, DSC1, and DSG1, were upregulated. This effect was the opposite of that observed in the control ACE +/+ THP-1 and C-domain-inhibited WT THP-1 macrophages where the same proteins were downregulated in comparison to the WT control.

Considering the functional enrichment overlap between the ACE-overexpressing control (ACE +/+ THP-1) and the C-domain-inhibited ACE-overexpressing (Lis–Trp + ACE +/+ THP-1) conditions, it is likely that the ACE C-domain plays a role within neutrophil degranulation regulation mediated by macrophage communication. This function is dysregulated in an increasing direction with C-domain inhibition. However, C-domain inhibition was also able to reduce glycolytic protein expression, significantly dysregulating glucose metabolism and downstream precursor metabolite and energy metabolism. Furthermore, the widespread RNA and splicing dysregulation across all comparisons suggests a role for ACE, emphasizing a non-classical function in signaling and gene expression. ACE overexpression was also confirmed to increase and significantly enrich cellular respiration, particularly aerobic pathway components, and TCA cycle components in human macrophages as previously identified in mice [34]. These same enriched terms were absent after Lis–Trp administration suggesting a direct role of ACE catalytic or signaling ability in regulating these processes. Some innate immunological enrichment was also present aside from neutrophil degranulation with ACE overexpression, specifically interleukin and cytokine signaling that are classically associated with Ang II expression rather than being independent of ACE functionality.

## 3. Discussion

Previous mouse model studies have revealed an altered, stronger immune response associated with increased ACE expression in macrophages and neutrophils [21,24,25,26,33,34,36,39]. ACEis have also been shown to impact other cell types [39,46], and these changes are not isolated to serum or endothelial ACE. It is, therefore, important to fully understand how immune cells are affected by these widely used medications. The present study provides a valuable resource in relation to the exploration of global proteomic and metabolic changes that occur in human macrophages caused by high ACE expression or domain-specific ACEi therapy. Data independent acquisition (DIA) mass spectrometry was used to identify global proteome changes and the associated enriched GO terms and reactome pathways in human ACE-overexpressing THP-1 macrophages. Furthermore, we assessed whether a C-domain-selective ACEi, Lis–Trp, could enter macrophages expressing intracellular ACE, and the impact of ACE C-domain inhibition on the myeloid cell proteome.

For human neutrophils, the ACEi ramipril has been shown to reduce bactericidal activity, but intracellular uptake has neither been measured nor confirmed [39,47]. In human macrophages, uptake of any ACEi has also not been confirmed despite evidence of their influence on macrophage function [47,48]. Treatment of THP-1 macrophages with 10 µM Lis–Trp resulted in a detectable increase in intracellular levels after 1 h, before decreasing 2 h post-treatment, whereas an upward trend of intracellular Lis–Trp was detected throughout the 2-h incubation period following 100 µM Lis–Trp treatment. Due to its low lipophilicity (cLogP = −80, ChemDraw version 16) and high total polar surface area (TPSA = 154, ChemDraw version 16), Lis–Trp has limited passive diffusion across membranes [41]. Similar to lisinopril, Lis–Trp could plausibly undergo active uptake via proton-coupled oligopeptide transporters or peptide carrier-mediated transport, resulting in low bioavailability (5.4% orally in rats) [49,50,51]. In the present study, <1% Lis–Trp was detected in THP-1 macrophages. A previous study observed a maximum of 10% uptake in the Caco-2 cell line after 10 µM lisinopril treatment [52]. Indeed, lisinopril requires active absorption through intestinal peptide carrier-mediated transporters, typically PEPT1 and PEPT2, but a mix of both active and passive uptake has been reported with an oral bioavailability of 25% [41,51,53,54].

Due to structural similarities, this same mechanism may be applied to Lis–Trp uptake in immune cells. However, THP-1 macrophages only express the peptide carrier PEPT2, and the peptide/histidine transporters PHT1 and PHT2 [55]. These transporters actively move amino acids and other hydrophilic molecules across cell boundaries [56,57,58], and high doses of Lis–Trp could, therefore, quickly exhaust cellular energy reserves, thus slowing antiport transport dramatically [54,55,59,60,61]. Due to a lack of saturation observed following the 100 µM treatment, we hypothesize that, at lower concentrations (10 µM), Lis–Trp is sufficiently removed from cells via efflux/transporters in the membrane, reaching an equilibrium. This system is then overwhelmed at 100 µM and is unable to maintain equilibrium, similar to the observations of Dumont et al. in *Escherichia coli* expressing AcrAB efflux pumps at high ciprofloxacin doses [59,61,62,63]. Alternatively, it is possible that Lis–Trp moves slowly into the THP-1 macrophages due to poor permeability, causing an increase over time at high concentrations. Lis–Trp may also be degraded and/or partially metabolized once internalized by macrophages due to high hydrolase levels, resulting in the observed decrease in intracellular Lis–Trp levels at a low dose. As with efflux pumps, high drug doses result in a maximum rate of enzymatic degradation and compound accumulation within cells. Lis–Trp may, therefore, require more time to reach its saturation point within macrophages, a phenomenon which is supported by the higher intracellular levels of Lis–Trp at a higher dose.

Having confirmed detectable Lis–Trp within THP-1 macrophages, we sought to validate ACE inhibition after uptake. Partial ACE inhibition was observed following Lis–Trp treatment in THP-1 macrophages, which was expected since Lis–Trp is C-domain-selective. The modified Z-phenylalanine-L-histidyl-L-leucine (ZFHL) ACE assay by Schwager et al. measures ACE activity regardless of domain [64,65]. The residual ACE activity observed for both 10 µM (≈31%) and 100 µM (≈43%) Lis–Trp treatments suggests that the N-domain remains active and capable of cleaving the Ang I analogue, ZFHL. Although partial ACE inhibition was observed, previous studies have indicated that partial inhibition, particularly of the C-domain, may negatively impact ACE-related immune function [20,21,39]. These effects should therefore be carefully considered in cases where patients are particularly vulnerable to infection or are immunosuppressed.

**Figure 6:**
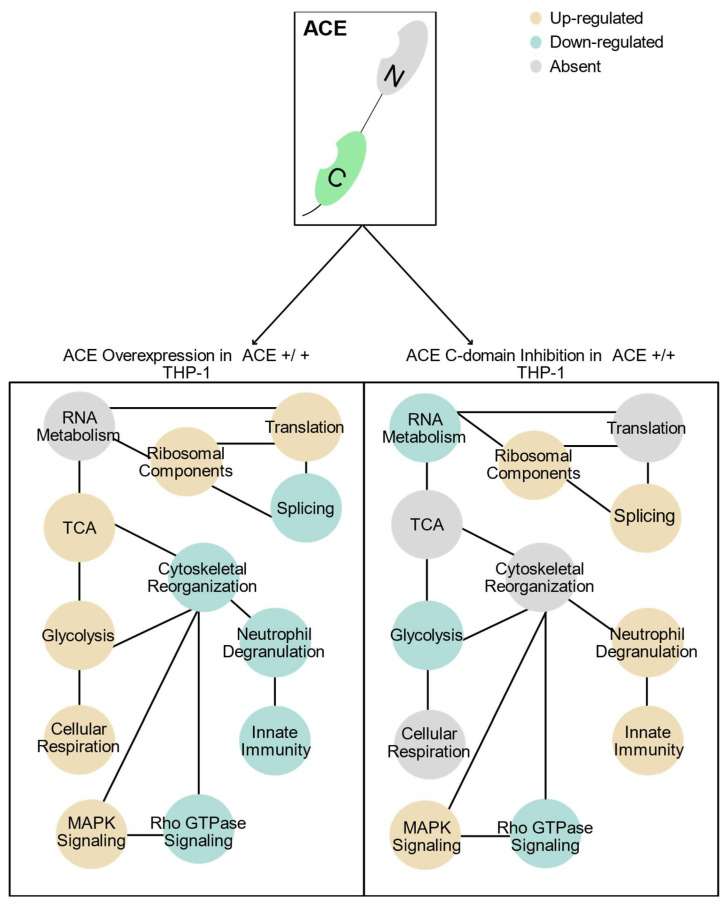
Summary of the functionally enriched biological process (BP) protein groups for ACE overexpression compared to ACE C-domain inhibition. Blue = downregulation, yellow = upregulation, and gray = absent functional enrichment during gene ontology (GO) and reactome analysis.

We next characterized the proteomic changes that are associated with inhibition of the ACE C-domain in ACE-overexpressing and WT THP-1 macrophages and the proteomic changes associated with ACE overexpression in comparison to WT THP-1 macrophages (Figure 6). We identified differentially expressed proteins in ACE +/+ THP-1 macrophages similar to those observed in ACE 10/10 murine macrophages. Importantly, ACE +/+ THP-1 macrophages had upregulated glycolytic and energy metabolism proteins in comparison to WT THP-1 macrophages and downregulated glucose metabolism-associated proteins with Lis–Trp treatment in ACE-overexpressing macrophages. ACE 10/10 murine macrophages have increased cellular ATP levels and an increased tricarboxylic acid (TCA) cycle and oxidative metabolic activities [34]. Having also used mass spectrometry and chemical analyses, Cao et al. [34] identified the increased TCA intermediates: citrate, isocitrate, succinate, and malate in ACE 10/10 murine macrophages. In the present study, three TCA proteins connected to TCA intermediates were identified and upregulated in ACE +/+ THP-1 macrophages and not in WT THP-1 macrophages, i.e., fumarate hydratase (FH), malate dehydrogenase 2 (MDH2), and dihydrolipoamide S-succinyltransferase (DLST). These enzymes are involved in the conversion of fumarate to malate, malate to oxaloacetate, and of 2-oxoglutarate to succinyl-CoA, respectively. We also observed functional enrichment and a significant increase in expression of the mitochondrial ATP synthase components ATP5F1A and ATP5F1B in ACE +/+ THP-1 macrophages compared to WT THP-1 macrophages. These pathways are associated with increased superoxide production and phagocytosis in macrophages and have also been observed in ACE 10/10 macrophages providing both improved energy metabolism and precursor components used in immune function [15,18,34].

The presence of immune-related proteins was expected, given that THP-1 macrophages are an immune cell type. The dysregulation of these proteins by Lis–Trp in ACE +/+ THP-1 macrophages would give insights into whether ACE overexpression can manipulate immune signaling in these cells. Although ACE +/+ THP-1 macrophages had functionally enriched immune system and innate immune-system-related proteins compared to WT THP-1 macrophages, the processes related to phagocytosis, MHC presentation, T cell regulation, and ROS production were not significantly enriched. The highly curated reactome-based functional analysis generated specific enrichment terms relevant to understanding ACE function in humans [66]. According to the reactome database, interleukin-12 and cytokine signaling, as well as neutrophil degranulation, were significantly functionally enriched pathways within ACE +/+ THP-1 macrophages, and the proteins associated with these immune functions were predominantly downregulated within the dataset. Bernstein et al. observed altered interleukin (IL-12β), cytokine (TNF), and complement system (C3) protein expression in the ACE 10/10 model, but further investigations are required to understand the importance of these findings in the context of ACE overexpression [19,28,67]. A strong immunological stimulation such as interferon-gamma (IFN-γ) would likely increase immunological functional enrichment and significantly upregulate immune-associated proteins in ACE +/+ and WT THP-1 macrophages. Importantly, it appears that, at rest, ACE overexpression in human macrophages does not overpower their immune regulatory systems, but may rather be priming macrophages through the preparation of precursor metabolites for a future immune response.

Interestingly, neutrophil degranulation was functionally enriched in ACE +/+ THP-1 macrophages as well as in cells treated with Lis–Trp compared to WT and untreated cells, respectively. Neutrophil degranulation has not previously been associated with ACE overexpression in macrophages, and this result, therefore, warrants further investigation, particularly as macrophage–neutrophil crosstalk remains understudied [68]. Neutrophils and macrophages are the first responders of the innate immune system, and, therefore, possess a strong arsenal against microbial intruders, including intracellular vesicles containing granules of antimicrobial proteases [69,70]. These granules are released, either extracellularly or intracellularly, in a controlled, sequential manner during degranulation. While it can protect the host from infection, extracellular degranulation can also cause tissue damage, which is why neutrophils require strong regulation [69,71,72]. In the present study, ACE overexpression provided a protective effect through macrophages, thus preventing the initiation of neutrophil degranulation. Cytoskeletal reorganization was functionally enriched in ACE-overexpressing cells, but it was absent in ACEi-treated cells compared to WT and untreated cells, suggesting that related proteins are dysregulated by both ACE overexpression and inhibition in macrophages. As part of the neutrophil degranulation mechanism, macrophage-regulated actin cytoskeletal reorganization occurs to allow granule translocation to the plasma and phagosomal membranes during exocytosis [68,72]. However, several degranulation and cytoskeletal proteins were downregulated in ACE +/+ THP-1 macrophages compared to WT macrophages, suggesting that macrophage crosstalk signaling can prevent degranulation without further stimulation, thus preventing host tissue damage. When the ACE C-domain was inhibited, neutrophil degranulation was enriched in both ACE +/+ and WT THP-1 macrophages, suggesting involvement of the C-domain in the regulation of this process. The associated proteins were significantly upregulated in Lis–Trp-treated ACE +/+ THP-1 macrophages compared to the control. A domain-selective ACEi such as Lis–Trp could, thus, be activating or inducing neutrophil degranulation and apoptosis, which was previously observed with lisinopril treatment in polymorphonuclear neutrophils (PMNs) by Miselis and colleagues [73]. Since ACEis cause different effects when given in vitro or orally, ACEis may have limited effects on circulating neutrophil function. Wysocki et al. [74] noted no hydrogen peroxide (H_2_O_2_) release in unstimulated PMNs, and no impact on ROS and H_2_O_2_ release in stimulated PMNs. In vitro, enalapril inhibited H_2_O_2_ release and the addition of captopril or enalaprilat also reduced neutrophil chemotaxis [74,75]. More recently, Cao et al. [39] provided evidence that ACE inhibition causes reduced neutrophilic action both in vivo and ex vivo. Although the focus of the present study was macrophages, it appears that ACE inhibition in macrophages could cause enhanced neutrophil degranulation and apoptosis, thus indirectly reducing neutrophil survival and function. Importantly, degranulation is central to neutrophil function and macrophages cooperate to attract neutrophils to the site of infection/inflammation. The associated mechanisms of degranulation rely heavily on neutrophil–macrophage crosstalk, but the opposite macrophage–neutrophil degranulation and communication mechanisms are unclear [76]. ACE’s ability to function as a signal transducer when bound by its inhibitors or substrates may be important in a macrophage-–neutrophil degranulation mechanism [77,78]. This is particularly pertinent in MAPK signaling when ACEis are present and highlights the roles of ACE that are independent of Ang II and its catalytic abilities [79,80,81].

In the present study, phosphorylation patterns and RNA metabolism were dysregulated and overrepresented across all experimental conditions. However, no direct influence on RNA splicing, translation, or transcriptional modification by ACE up- or downregulation has been previously observed, despite large-scale modifications of cellular function in ACE 10/10 and NeuACE models. RNA binding proteins, including ribosomal interactors, were particularly enriched within our dataset, with 47 and 37 proteins identified in untreated and Lis–Trp-treated ACE +/+ THP-1 macrophages, respectively. Transcriptomic analysis could provide valuable insights into the similarities of these protein expression profiles and elucidate the changes in gene expression across ACE overexpression models. Altered RNA metabolism and transcriptional modifications may also pinpoint RNA–protein interactors of interest. Given the functional enrichment of broad RNA-related terminology that is associated with both ACE overexpression and ACE inhibition in the present study, a more RNA focused study approach may be warranted in the future.

MAPK and Rho GTPase functional enrichment were also associated with ACE overexpression. These terms hint at a modified cellular signaling within ACE +/+ THP-1 macrophages via phosphorylation. ACE, when bound by ACEis or substrates, participates in endothelial MAPK and AP1 signaling where it mediates downstream signaling cascades [77,78]. The binding of ACE to inhibitors, for instance captopril and ramiprilat, results in cytoplasmic Ser^1270^ phosphorylation by casein kinase 2 (CK2) activation, leading to c-Jun N-terminal kinase (JNK) or extracellular signal-regulated kinase 1/2 (ERK1/2) signaling activation, modulating inflammatory gene expression and vascular remodeling [78,81,82]. It is hypothesized that an as yet unidentified ACE substrate is responsible for the improved immune responses seen in ACE 10/10 and NeuACE mice [19]. Consequently, the substrate may structurally resemble an ACEi, since it modulates cell phosphorylation patterns in place of undergoing ACE catalysis. Previously, increased MAPK phosphorylation and superoxide production has been observed in NeuACE neutrophils [39]. In such a study, ACEi reduced phosphorylation levels despite high ACE expression in this model, whilst ARBs had no significant effect. In this investigation, in Lis–Trp-treated WT THP-1 macrophages, protein tyrosine kinase 6 (PTK6)-regulated transforming protein RhoA (RHOA) and adapter molecule crk (CRK) were markedly increased compared to the control, supporting previous observations of increased MAPK phosphorylation in ACE expressing cells after ACE inhibition. These proteins also mediate cell migration and adhesion, both of which are important processes in macrophages. These same components could also play a role in neutrophil degranulation regulation via macrophage communication. MAPK/ERK signaling also induces ACE upregulation and ACE2 downregulation, which was previously observed with long-term ACE inhibition [82,83]. It is, thus, possible that the levels of ACE may increase over time, gradually inducing the benefits associated with its increased expression, particularly in immune cells. Whilst MAPK/ERK signaling and inflammatory gene expression can be both Ang-II-dependent and independent, Ang II also activates Rho GTPase signaling as part of a cardiovascular remodeling [84]. Rho GTPase controls both cell adhesion and migration by cytoskeletal reorganization [84,85,86], which is a functionally enriched GO BP term in the ACE +/+ THP-1 macrophage cell line of this study. Although pathway dysregulation was observed in macrophages with increased ACE expression, MAPK functional enrichment was, unexpectedly, absent upon Lis–Trp treatment in ACE +/+ THP-1 macrophages. However, MAPK, Rho GTPase, and RAS GTPase functional enrichment persisted with the Lis–Trp treatment of WT THP-1 macrophages, supporting previous evidence of heightened kinase activation with ACEi administration targeting both plasma and endothelial ACE [78,79,81,82].

In the current body of work, we aimed to survey the global proteome of a human ACE-overexpressing macrophage cell line to ascertain if ACE overexpression leads to favorable immune outcomes in human macrophages. This study is the first to present such results and highlights the need for further targeted and mechanistic investigations, particularly into the TCA and oxidative metabolism components associated with the increased ROS and cellular ATP in ACE 10/10 murine macrophages. We hypothesize that phagocytosis is impacted in the ACE +/+ THP-1 cell line, since both the TCA cycle and the energy metabolism-associated proteins were functionally enriched and upregulated by ACE overexpression within our dataset. Based on these exploratory results, we conclude that it is worthwhile pursuing the mechanism of ACE overexpression and its potential as an alternative therapy in disease management. Indeed, ACE overexpression in murine macrophages is promising for the treatment of resistant microbial and cancerous conditions. This may be used as an alternate immunotherapy, whereby macrophages are genetically altered to express high baseline ACE levels, thus activating a novel signaling cascade capable of inducing a strong immune response to disease.

## 4. Materials and Methods

### 4.1. Cell Culture Conditions

The human monocytic cell line, THP-1 (ATCC^®^ TIB-202^TM^, Manassas, VA, USA), was grown in Roswell Park Memorial Institute (RPMI) 1640 medium (Merck, Darmstadt, Germany), supplemented with 10% (*v*/*v*) fetal bovine serum (FBS), 10 mM HEPES (4-(2-hydroxyethyl)-1-piperazineethanesulfonic acid), and 0.2 mM L-glutamine. Standard culturing conditions at 37 °C and 5% CO_2_ in a humidified incubator were used. Cells were monitored and aseptically maintained to ensure constant cell morphology and no contamination at a density of 1 × 10^6^ cells/mL.

#### THP-1 Differentiation into Macrophages

To differentiate monocytes into macrophages, 25 ng/mL PMA (Sigma, St. Louis, MO, USA) was added to 2 mL of cell suspension between 2 × 10^5^ cells/mL and 1 × 10^6^ cells/mL in a six-well culture plate and 10 mL culture dish for Lis–Trp uptake and ACE activity analysis, respectively. THP-1 monocytes were differentiated for 24–48 h until 90% adherent. Adherent THP-1 macrophages were washed with cold 1× phosphate-buffered saline (PBS) three times before the addition of PMA-free 10% RPMI medium and a 24-h incubation to allow for normal gene expression after PMA stimulation.

### 4.2. Lisinopril–Tryptophan Uptake Assessment

#### 4.2.1. Lisinopril–Tryptophan Treatment and Cell Lysis

Following PMA differentiation, the adapted method of Chen et al. [87] was used for THP-1 ACE inhibition, cell lysis, and inhibitor quantification. Briefly, three time points were recorded for the analysis of intracellular Lis–Trp uptake: zero-time, one hour, and two hours. Lis–Trp [42] was added at either 10 µM or 100 µM final concentration and each time point was incubated before lysis.

Lysis was conducted on ice to minimize protease activity. Cells were washed with cold 1× PBS three times before adding 1 mL of 5 mM EDTA (ethylenediaminetetraacetic acid) to gently lift and lyse the cells. After 15 min, the remaining cells were scraped from the plate and the resulting cell lysate collected. During EDTA incubation, a Trypan Blue exclusion assay was performed within five minutes to quantify cell numbers and evaluate cell viability. Cell lysates for ACE activity measurement were prepared with Triton X-100 lysis buffer as EDTA inhibits ACE [88,89]. After lysis, the protein concentration was determined using the Bio-Rad Bradford Reagent assay [90] (Bio-Rad, Hercules, CA, USA).

As a background for protein complexity during mass spectrometric analysis, a 50 mL bulk culture of THP-1 macrophages was prepared at a density of 1 × 10^6^ cells/mL. Cells were centrifuged at 300× *g* and washed three times with cold 1× PBS before lysing in 50 mL of 5 mM EDTA.

#### 4.2.2. Mass Spectrometric Analysis of Intracellular Lisinopril–Tryptophan

Each experimental condition was cultured in triplicate, and each cell lysate was analyzed in triplicate using an AB Sciex 5500 QTrap^®^ mass spectrometer (Framingham, MA, USA).

##### Sample Preparation and Extraction

Lysed samples were vortexed to ensure homogeneity before acetonitrile (ACN) extraction using 100 µL cell lysate and 200 µL ice-cold ACN containing 5 nM Verapamil as an internal standard. Each sample supernatant was added to a 96-well plate for liquid chromatography tandem mass spectrometry (LC-MS/MS) analysis, including calibration and quality control standards. Standards consisted of bulk macrophage lysate spiked with Lis–Trp over a range of 1–3125 ng/mL. Standards were analyzed together with samples after ACN extraction to create a standard curve.

##### Liquid Chromatography Tandem Mass Spectrometry (LC-MS/MS) Analysis

A Sciex 5500 QTrap^®^ mass spectrometer equipped with an electrospray ionization (ESI) source in positive ionization mode and an Agilent 1290 Rapid Resolution HPLC (Santa Clara, CA, USA) were used for the analysis. Multiple reaction monitoring (MRM) was used to measure transitions of the protonated molecular ion of Lis–Trp at 495 *m/z* and its product ions 84 *m/z* (quantifier) and 291 *m/z* (qualifier). The Verapamil transition was at 455 → 303 *m/z*. HPLC separation was conducted on a Poroshell C18 (50 × 4.6 mm, 2.6 µm) column (Agilent, Santa Clara, CA, USA), with 0.1% formic acid (FA) as the aqueous mobile phase (A) and 0.1% FA in ACN as the organic mobile phase (B). Elution was achieved with a linear 10–100% solvent B gradient over 1.8 min at a flow rate of 0.7 mL/min, starting 0.2 min after sample injection. The gradient was held at 100% B for one minute before re-equilibration of the column at 10% B for 2.9 min. A six-minute needle wash was performed in between each sample, consisting of water, ACN, methanol (MeOH), and isopropanol at a 30:30:30:10 ratio.

### 4.3. ACE Activity

The enzymatic ZFHL (Bachem AG, Bubendorf, Switzerland) assay was performed using the protocol by Schwager et al. [64]. Briefly, 5 µL of cell lysate was incubated in 30 µL of 2 mM ZFHL at 37 °C for 15 min. An adduct was formed by the addition of 0.28 M NaOH-7mM-*o*-pthaldialdehyde solution and incubated at room temperature for 10 min. The addition of 3 M hydrochloric acid (HCl) halted the reaction, and the fluorescence was measured using a λ_excitation_ of 360 nm and λ_emission_ of 485 nm (Varian Cary Eclipse, Agilent, Santa Clara, CA, USA). To convert fluorescent units into milliunits (mU) ACE activity, an L-histidyl-L-leucine (HL) (Merck, Darmstadt, Germany) standard curve was generated. One unit of ACE activity is defined as 1 mole of HL produced/minute/mL by ACE at 37 °C.

### 4.4. Global Proteome Analysis via Triple Time-of-Flight (TOF) Mass Spectrometry

#### 4.4.1. Macrophage Treatment for Proteomic Analysis

The ACE +/+ cell line, expressing full-length human somatic ACE, was created using a lentivirus vector system by Systems Biosciences (SBI), Palo Alto, CA, USA, and lysates thereof were gifted by the K. Bernstein laboratory (Cedars-Sinai Medical Centre, Los Angeles, CA, USA). The ACE +/+ cell line was created by stably transfecting the THP-1 cell line with human ACE in the SBI CD710B-1 vector containing a murine stem cell virus (MSCV) promoter. An empty vector THP-1 cell line was similarly created [27]. ACE +/+ and empty vector (denoted as WT) THP-1 monocyte/macrophages were cultured under standard conditions with the addition of 1 µg/mL puromycin co-culture for vector selection. The ACE C-domain inhibitor, Lis–Trp, was added to a subset of ACE +/+ and WT THP-1 macrophages at 50 µM for 24 h before lysis in radioimmunoprecipitation assay (RIPA) buffer, supplemented with a protease inhibitor cocktail. Three biological replicates per experimental condition were collected.

##### Sample Preparation

Gifted ACE +/+ THP-1 and WT THP-1 macrophage lysates were subjected to acetone:methanol precipitation at a ratio of 8:1. All cell lysates were transferred to LoBind^®^ Eppendorf tubes (Eppendorf, Hamburg, Germany), at nine parts ice-cold acetone:methanol to 1 part lysate. Proteins were precipitated by incubating at −20 °C overnight. The precipitated proteins were centrifuged at 4000× *g* for 10 min, and the pellet was washed with ice-cold 80% acetone and airdried. Pellets were then resuspended in denaturation buffer (6 M urea, 2 M thiourea in 10 mM Tris-HCl, pH 8.0).

Protein concentration was quantified using the Bio-Rad Bradford reagent assay [90]. Duplicate samples were spectrophotometrically quantified at an absorbance of 595 nm using an iMark^TM^ Microplate Absorbance Reader (Bio-Rad, Hercules, CA, USA). The bovine serum albumin (BSA) standard curve was prepared in triplicate over a range of 0–2 mg/mL.

##### In-Solution Digestion

For each 5 µg total digest, the reducing agent dithiothreitol (DTT) was added to a final concentration of 3 mM and incubated for 20 min at room temperature (RT). The alkylating agent iodoacetamide (IAA) was then added to each sample at a final concentration of 15 mM and incubated in the dark for 20 min, at RT. Samples were then diluted 5× with 50 mM ammonium bicarbonate to dilute the urea to below 1 M and ensure a pH of 8.0. Trypsin was added at a ratio of 1:100 µg enzyme:protein and incubated overnight at 30 °C. Digestion was halted with the addition of 0.5% FA.

##### Desalting

EvoTips (Evosep Biosystems, Odense, Denmark), disposable C18 trap columns, were used for offline desalting and elution of peptides directly into the LC-coupled Sciex TripleTOF^®^ 6600 mass spectrometer (Sciex, Framingham, MA, USA) according to the manufacturer’s instructions. Tips were rinsed using solvent B (ACN, 0.1% FA) and centrifuged at 800× *g* for 1 min, and then conditioned by soaking with propanol until the tips were pale white. Solvent A (2% ACN, 0.1% FA) was added to each tip and centrifuged at 800× *g* for 1 min to equilibrate, after which a 1 µg acidified peptide sample was loaded into each tip and centrifuged once more. The tips were washed with solvent A and centrifuged again, and then wetted by the addition of 100 µL of solvent A and centrifuged at 800× *g* for 10 s prior to mass spectrometry (MS) analysis.

#### 4.4.2. Triple Time-of-Flight (TOF) Sequential Window Acquisition of all Theoretical Mass Spectra (SWATH^®^) Mass Spectrometry

Liquid chromatography was performed on an Evosep One LC coupled to a Sciex TripleTOF^®^ 6600. Peptides were separated using the pre-programmed 40 sample per day (SPD) method at a column temperature of 40 °C using the recommended 15 cm, 75 µm column packed with 1.9 µm solid core beads. LC solvent buffer A (2% ACN and 0.1% FA) and buffer B (ACN and 0.1% FA) were used. The OptiFlow source was used with the nanoprobe set to 250 °C and a 3000 V spray voltage.

The Sciex 6600 was operated in positive mode using SWATH acquisition comprising a variable window scheme with 120 windows or minimum 3 *m/z* and 1 *m/z* overlap. The MS1 and MS2 fill time was set to 250 ms and 15 ms, respectively, giving an approximately 2 s cycle time.

#### 4.4.3. Data Processing and Clean-Up

Using data independent acquisition neural network (DIA-NN) software (version 1.8.1) [91], raw data were processed to identify and normalize label-free quantification values matching against a pre-generated UniProtKB human proteome spectral library. The following settings were used as part of the database search: 1 missed cleavage, peptide lengths of 7–30 amino acids, and a mass range of 300–1150 *m/z* with cysteine as a variable modification. The resultant DIA-NN protein matrices were filtered to remove contaminants. Quantification data was log2 transformed using Perseus (version 2.0.7.0) [92,93]. Values were considered to be valid if a protein was present in two of the three replicates in any group to account for the overexpression conditions in the ACE +/+ THP-1 macrophages.

### 4.5. Statistical Analysis

Data analysis was conducted in Perseus (version 2.0.7.0) [92,93] and RStudio (version 2023.06.0 + 421, RStudio, Boston, MA, USA). A student’s two sample t-test was applied to identify differentially expressed proteins between conditions, and a *p*-value ≤ 0.05 was considered to be statistically significant. The comparisons tested are shown in Figure S4.

During Lis–Trp uptake assessment, ACE activity, protein concentration, and cell counts were recorded as mean values ± standard deviation (SD) for the total cell lysate samples. Measured intracellular Lis–Trp concentrations were normalized against cell counts for each time point and treatment concentration, with outliers greater than 2 × SD excluded. Lis–Trp concentrations were normalized to cell count, as cells may express different levels of protein and protein recovery is not 100%. Box plots, bar, and line graphs were all generated using RStudio.

### 4.6. Functional Enrichment and Network Analysis

To generate protein–protein interaction (PPI) networks, Search Tool for the Retrieval of Interacting Genes/proteins (STRING) within Cytoscape (version 3.10.2) [94,95] was used for the significant differentially expressed proteins with *p* ≤ 0.05 and log2(Fold-Change) or log2FC ≥ |1.5|. The interaction confidence was set to medium (0.4), and line or edge thickness was used to indicate the confidence score of the interaction. Log2FC was continuously mapped to protein nodes, with blue representing downregulation and yellow representing upregulation. The shade of the node was attributed to the FC level, where a darker color indicated a larger FC. No additional interactors were permitted during network generation, and an MCL algorithm [96] with an inflation parameter of four was applied to identify highly connected protein complexes and biological pathways within the global network.

Following network generation, a functional enrichment analysis was performed using stringApp [97] within Cytoscape [95]. Gene ontology (GO) term enrichment for biological processes (BP) and reactome was analyzed [66] using a hypergeometric test with Benjamini–Hochberg FDR correction identifying significantly overrepresented proteins (*p* ≤ 0.05).

## 5. Conclusions

Using Triple-TOF SWATH mass spectrometry, we were able to identify dysregulated biological pathways within a human ACE-overexpressing THP-1 macrophage cell line. These pathways included proteins associated with neutrophil degranulation, TCA intermediates, RNA metabolism, and innate immunity. Increased cellular respiration stipulated by upregulated TCA components, glucose metabolism-associated proteins, and ATP synthase components in the human ACE +/+ THP-1 macrophages and ACE 10/10 murine macrophages are encouraging regarding the use of increased ACE expression as an alternative immunotherapy and means of enhancing the immune response. The present study also provides evidence of Lis–Trp uptake in human macrophages being capable of partially inhibiting ACE activity, predominantly the C-domain. Importantly, we show that Lis–Trp is capable of influencing ACE activity and, thus, significantly alter the THP-1 proteome. Additionally, we uncovered novel ACE influence over cellular splicing, transcription, and translation linked to ACE overexpression in human macrophages which is up-regulated with ACE C-domain inhibition. These changes have not previously been observed in the ACE 10/10 murine model. However, C-domain-selective Lis–Trp treatment ameliorated the altered energy-associated glucose and TCA protein expression profiles of ACE +/+ THP-1 macrophages, a similar observation having been made in murine ACE 10/10 macrophages and NeuACE neutrophils treated with ACEis, strengthening the C-domain’s role in the improved immune state. ACE hyperexpression and C-domain inhibition also altered cellular signaling via phosphorylation cascades. In particular, the MAPK, Ras GTPase, and Rho GTPase pathways were functionally enriched after C-domain inhibition. With large-scale cellular signaling and cytoskeletal and protein reorganization evident with ACE overexpression in human macrophages, an interesting and challenging road lies ahead with respect to our understanding of how this altered metabolic state benefits immunity.

## Data Availability

The raw data supporting the conclusions of this article will be made available by the authors upon request. The mass spectrometry proteomics data have been deposited in the ProteomeXchange Consortium [98] via the PRIDE [99] partner repository with the dataset identifier PXD052292.

## Supplementary Materials

The following supporting information can be downloaded at: https://www.mdpi.com/article/10.3390/ijms25137055/s1.
