# Supplementary figures and images for: Proteomic Analysis of Human Macrophages Overexpressing Angiotensin-Converting Enzyme
Source: Int J Mol Sci. 2024 Jun 27;25(13):7055. doi: 10.3390/ijms25137055 (PMC11240931; doi:10.3390/ijms25137055)

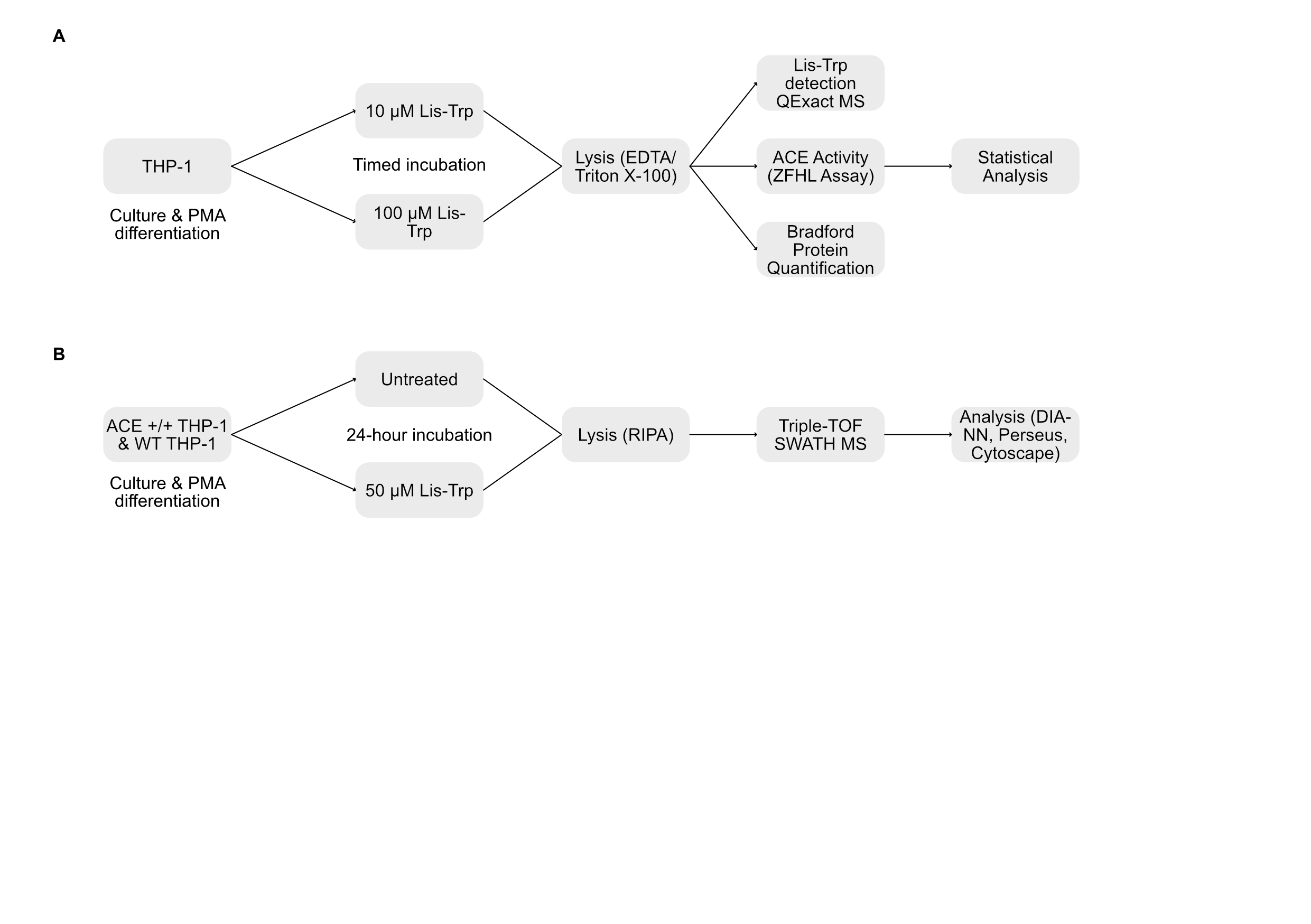

Supplement: Supplementary file 1 [file ijms-25-07055-s001.zip › Figure S1_Workflow.tiff]

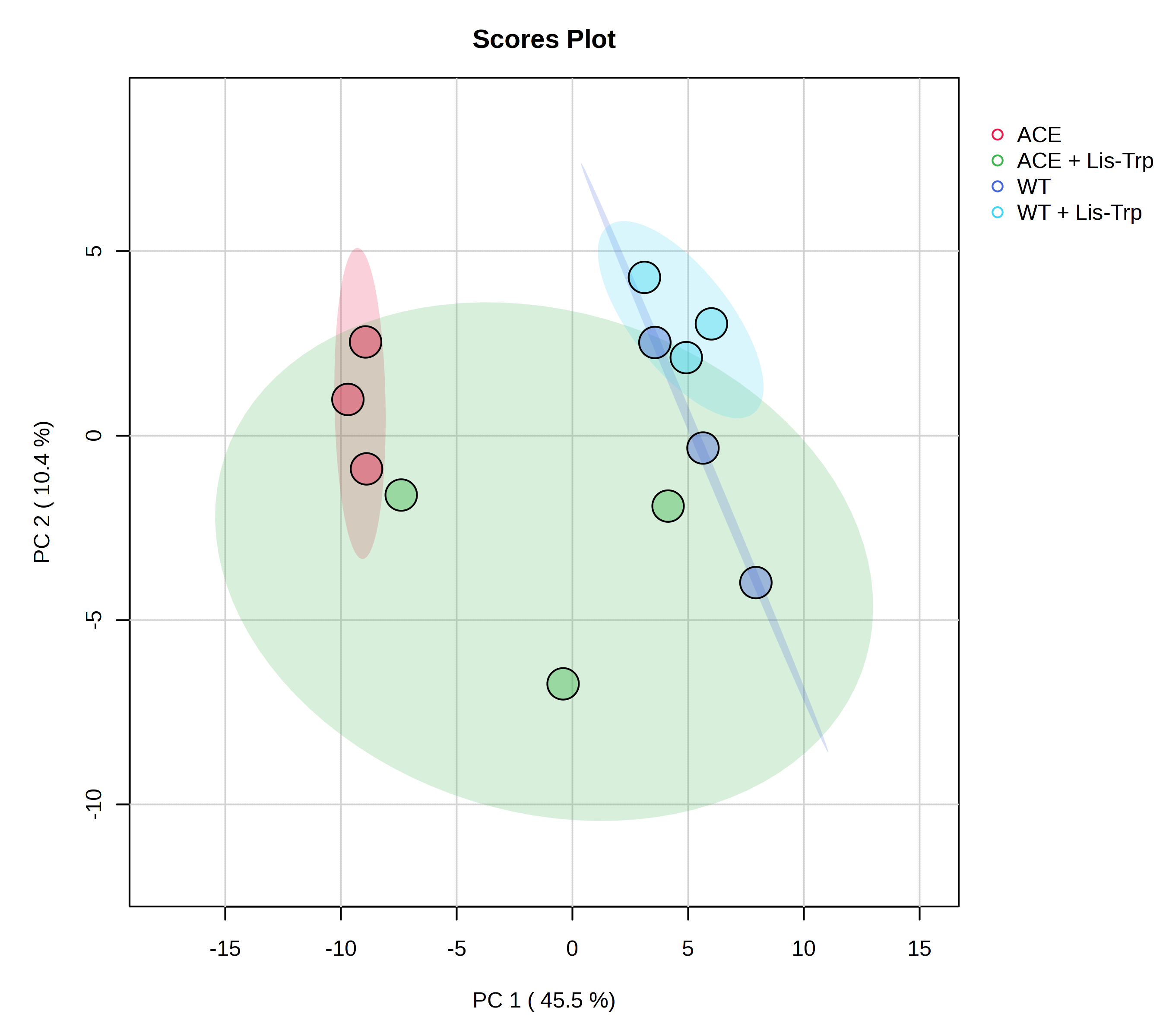

Supplement: Supplementary file 1 [file ijms-25-07055-s001.zip › Figure S2_PCA_300dpi.png]

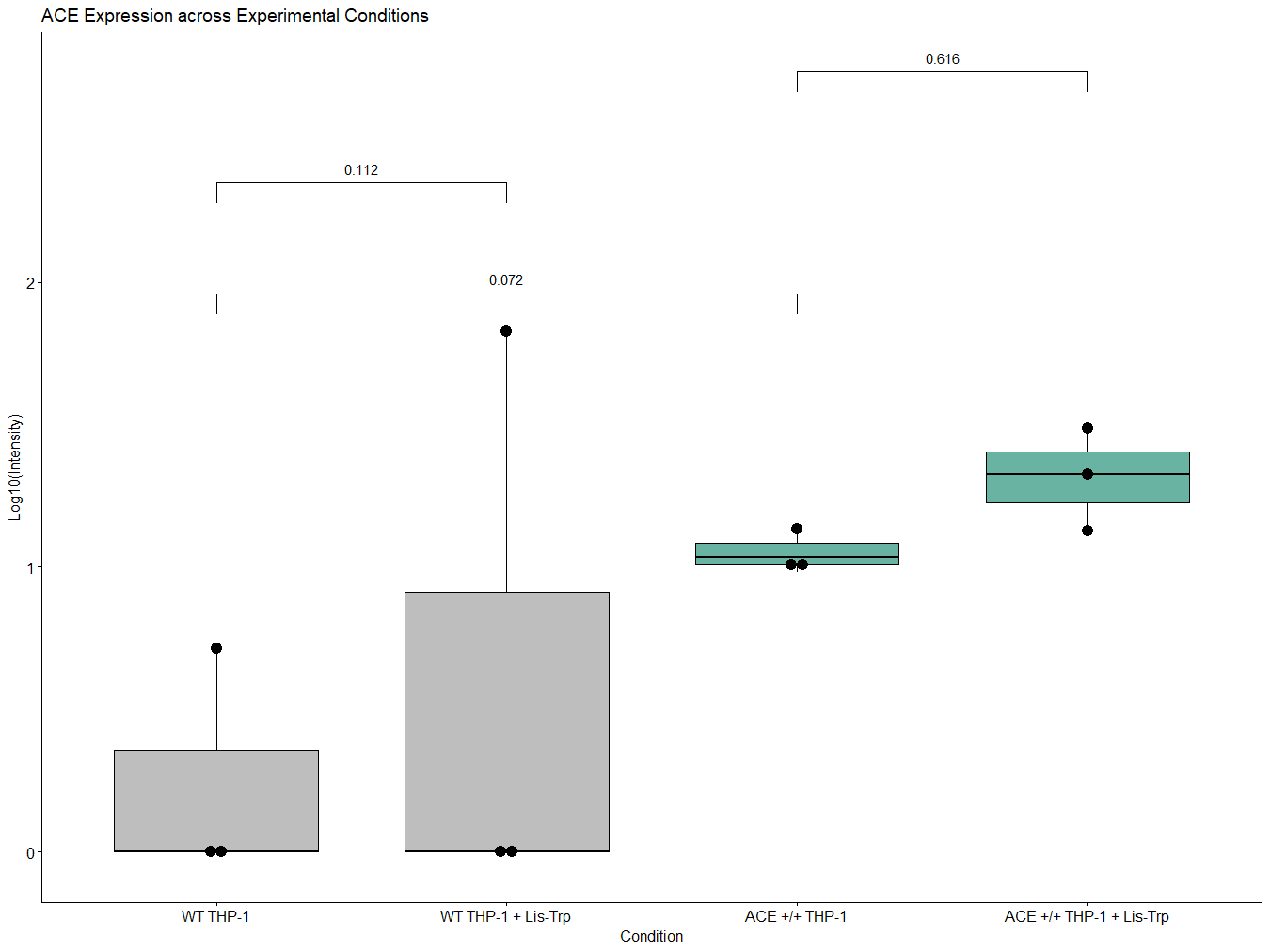

Supplement: Supplementary file 1 [file ijms-25-07055-s001.zip › Figure S3_ACE Ttest.tiff]

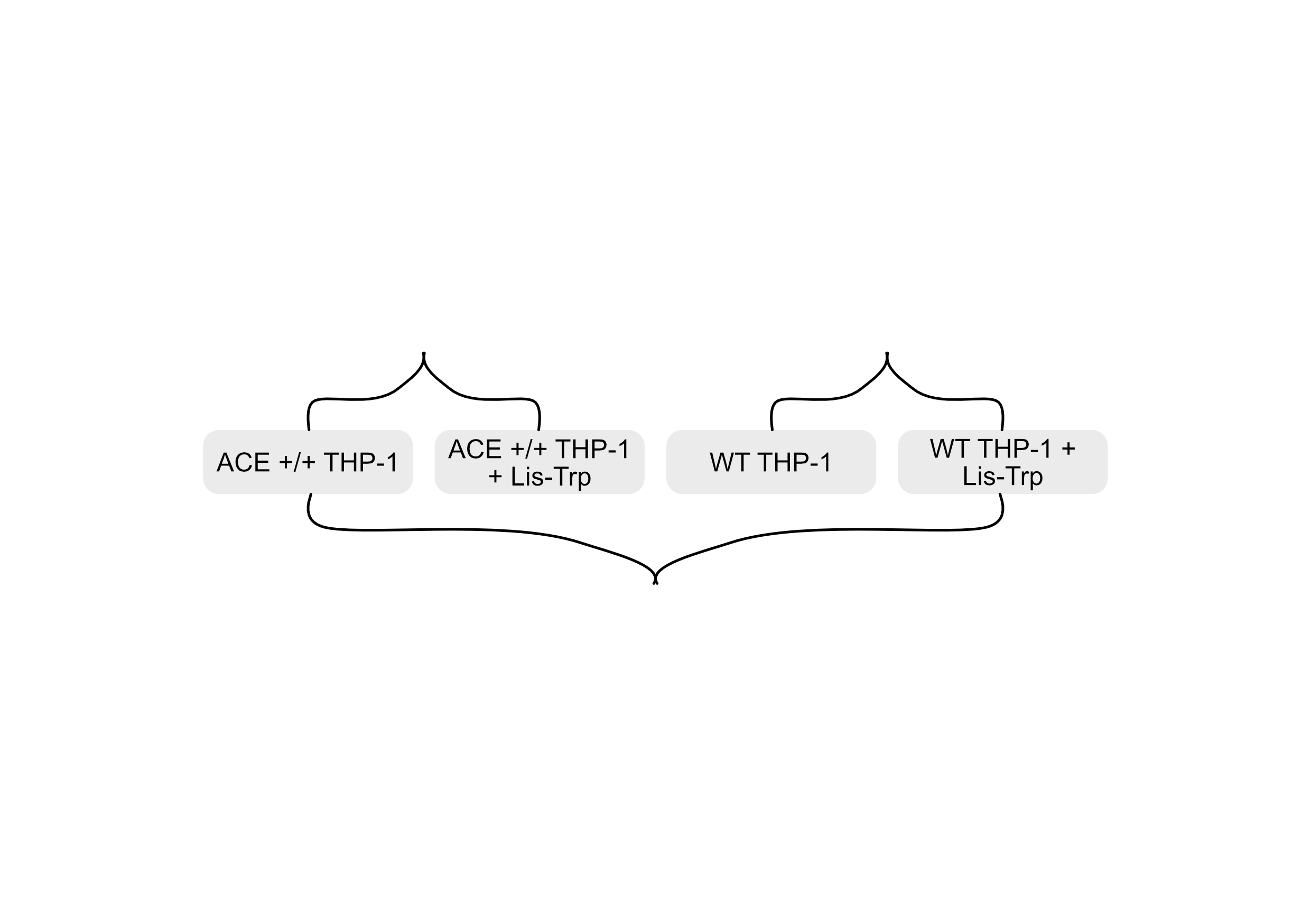

Supplement: Supplementary file 1 [file ijms-25-07055-s001.zip › Figure S4_Test Comparisons.tiff]
